# DICER1 Mutation-Associated Follicular Thyroid Tumor With Bizarre and Pleomorphic Nuclei

**DOI:** 10.7759/cureus.83644

**Published:** 2025-05-07

**Authors:** Mai Thy Tran, Hunter L Monroe, John A Ozolek, Reima El Naili

**Affiliations:** 1 Pathology and Laboratory Medicine, West Virginia University School of Medicine, Morgantown, USA; 2 Pathology, West Virginia University, Morgantown, USA; 3 Pathology, Anatomy and Laboratory Medicine, West Virginia University School of Medicine, Morgantown, USA

**Keywords:** anaplastic thyroid carcinomas, bizarre nuclei, dicer1 mutation, follicular thyroid carcinoma (ftc), thyroid carcinoma

## Abstract

Thyroid carcinomas comprise diverse subtypes, each characterized by distinct histomorphologies, molecular profiles, and prognoses. Follicular thyroid carcinoma (FTC) is a differentiated thyroid cancer demonstrating vascular and/or capsular invasion, while anaplastic thyroid carcinoma (ATC) is an aggressive and undifferentiated form with an exceedingly dismal prognosis. Thyroid carcinomas also can exhibit mixed histologic features, complicating the diagnosis and management of these entities. *DICER1 *germline and somatic mutations are associated with multiple neoplasms, including thyroid neoplasms along a morphological spectrum from benign, differentiated malignant carcinomas, and poorly differentiated carcinomas, suggesting a role for these mutations in tumor dedifferentiation and progression. We present a 31-year-old female with a large encompassing thyroid tumor harboring a somatic *DICER1* "hotspot" mutation (p.E1705K) displaying predominantly follicular microarchitecture, bizarre and pleomorphic nuclear features, yet without overt invasive or poorly differentiated features and low proliferation indices. While poorly differentiated follicular thyroid carcinomas have been associated with *DICER1 *mutations, the morphology present in this tumor, to our knowledge, has not been reported in *DICER1-*associated thyroid neoplasms. This case continues to expand the morphological spectrum of thyroid tumors with *DICER1 *mutation and again raises questions about the role of *DICER1 *in thyroid tumor morphology and behavior.

## Introduction

Thyroid carcinoma consists of a heterogeneous group of neoplasms with diverse biological behaviors and clinical outcomes, ranging from well-differentiated tumors with favorable prognoses to poorly-to-undifferentiated forms with highly aggressive behavior and poor outcomes. Follicular thyroid carcinoma (FTC) is typically classified as a differentiated thyroid cancer with a generally favorable prognosis. Diagnostically, follicular architecture and vascular and/or capsular invasion need to be observed, but it typically lacks high-grade features [1,2]. Conversely, anaplastic thyroid carcinoma (ATC) represents the most aggressive form of thyroid cancer, manifested by its rapid course and low overall survival rates. ATC frequently presents with a solid growth pattern, pronounced cytological atypia, a high mitotic rate, extensive necrosis, and frequent lymphovascular invasion. The stark presentation of this entity is further exacerbated by its known resistance to conventional therapeutic approaches [1]. FTC commonly harbors *RAS *mutations and PAX8-PPARG fusions, activating the PI3K/AKT pathway and driving tumor progression. In contrast, poorly differentiated thyroid carcinoma (PDTC) and ATC frequently exhibit TERT promoter, *TP53, RAS, and BRAF V600E *mutations, contributing to aggressive behavior [1,2].

*DICER1 *mutations, crucial in the microRNA (miRNA) processing pathway and post-transcriptional regulation of gene expression, have been identified across a spectrum of thyroid neoplasms, including neoplasms of follicular differentiation (benign and malignant), papillary carcinomas, thyroblastomas, poorly-differentiated carcinomas, and anaplastic carcinomas, suggesting a role in tumorigenesis and potential dedifferentiation [3, 4]. Thyroid tumors harboring *DICER1 *mutations also typically follow the clinical behavior associated with the type of thyroid tumor morphology. We present this case of a *DICER1*-associated thyroid neoplasm that clinically and by nuclear morphology seemed to represent a very aggressive tumor yet lacked overt signs of invasion, demonstrated low mitotic activity and proliferation, and had predominantly follicular architecture. 

## Case presentation

**Figure 1:**
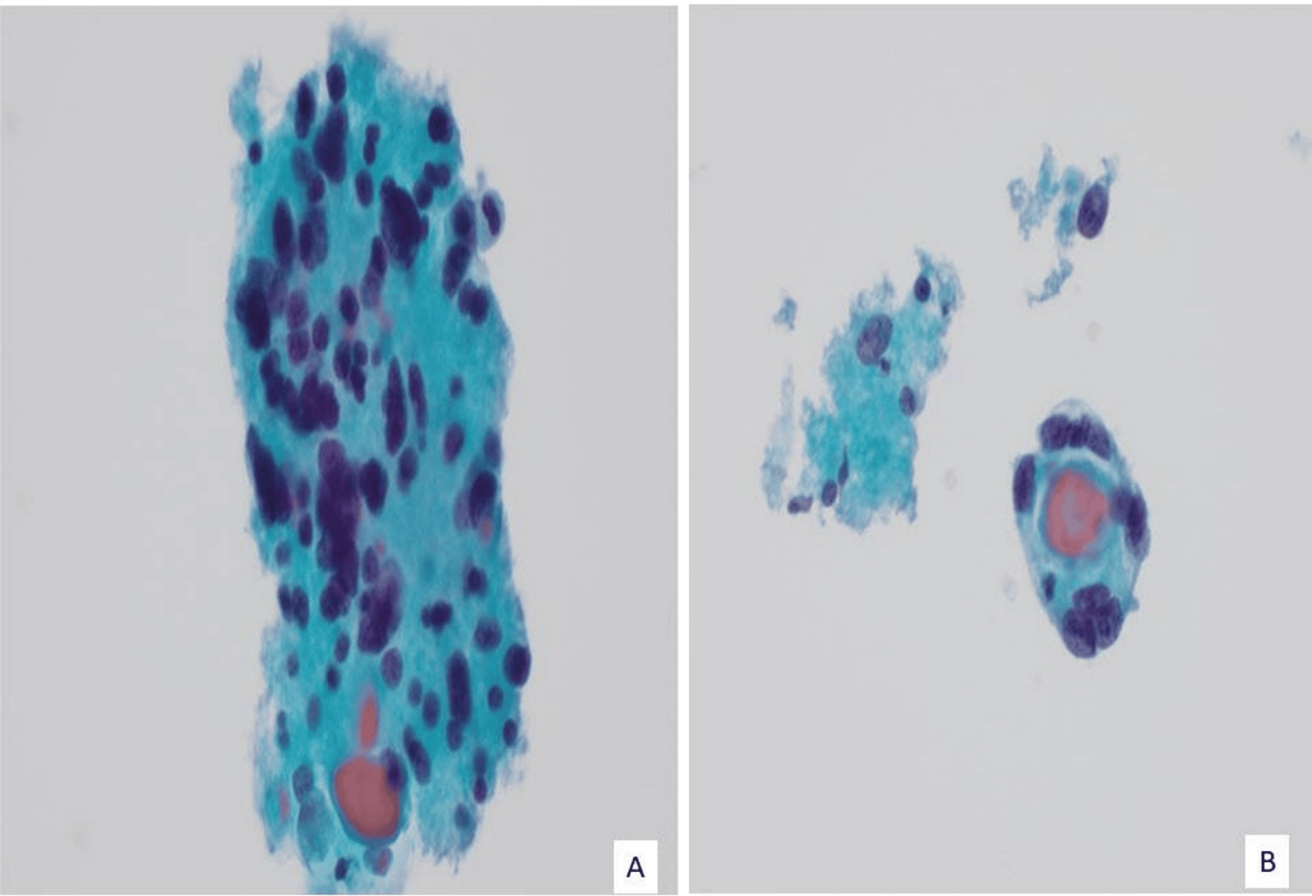
Fine-needle aspiration. A. Clusters of tumor cells with marked nuclear pleomorphism, hypochromasia, and clumped chromatin and occasional multinucleated cells in a background of colloid (H&E, 40X).  B. Tumor cells with microfollicular pattern (H&E, 40X).

A 31-year-old Caucasian female presented with left neck swelling first noticed during the third trimester of her pregnancy. Family history was notable for lymphoma, colon cancer, and throat cancer among her grandparents, but no known history of thyroid or other endocrine malignancies or radiation exposure. Ultrasound examination revealed a 6.2 x 2.8 x 4.2 cm mass in the left thyroid lobe that was isoechoic to slightly hypoechoic. Laboratory tests, including thyroid-stimulating hormone (TSH) and free thyroxine (T4), were within the normal range. Fine needle aspiration (FNA) of the left thyroid nodule demonstrated loosely clustered cells with marked hyperchromasia and nuclear pleomorphism, along with a variable microfollicular architecture within a colloid-containing background, diagnosed as positive for malignancy, thyroid carcinoma (Bethesda category VI), consistent with poorly differentiated thyroid carcinoma (Figure 1). Molecular analysis of the FNA material using Afirma® Genomic Sequencing Classifier and Xpression Atlas next-generation sequencing (NGS) (Veracyte Inc., Austin, USA) identified a *DICER1* "hotspot" mutation (p.E1705K, c.5113G>A). Invitae germline testing (Invitae Corp., San Francisco, USA) was performed and excluded a germline *DICER1 *mutation. FoundationOne CDx molecular test (Foundation Medicine Inc., Boston, USA) also shows a frameshift mutation in *TP53* N30fs*14 without microsatellite instability, and the tumor was negative for *BRAF V600E*, *RET/PTC, BRAF, *and *KRAS*. The patient underwent total thyroidectomy, which intraoperatively demonstrated a large friable tumor that extended behind the trachea, requiring extensive dissection from the adventitia of the esophagus and posterior tracheal attachments.

**Figure 2:**
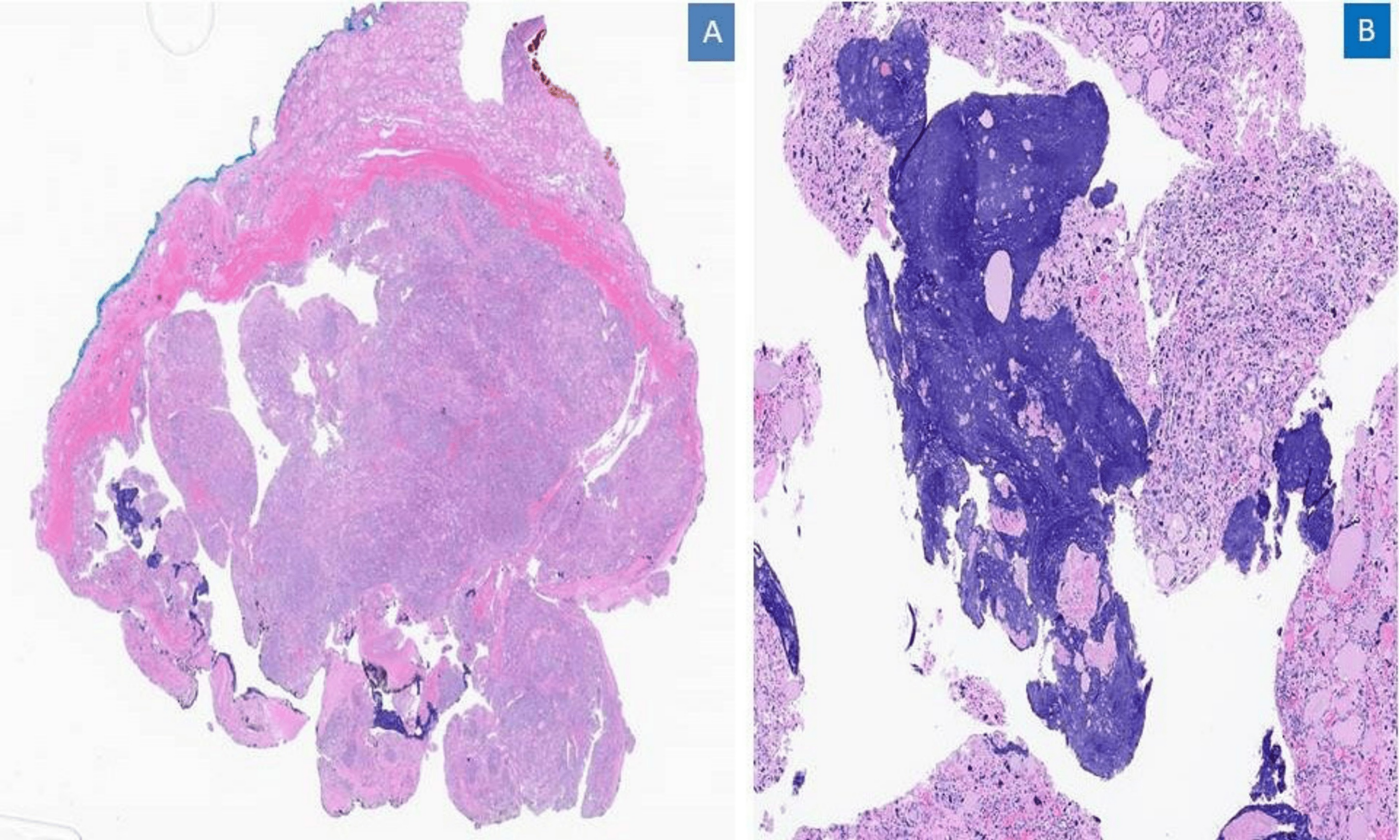
Tumor capsule A. Lower power image showing cellular tumor with intact irregular capsule(H&E 10X), B. Dark blue areas signified presumably smudged chromatin (Azzopardi effect, H&E 50X).

**Figure 3:**
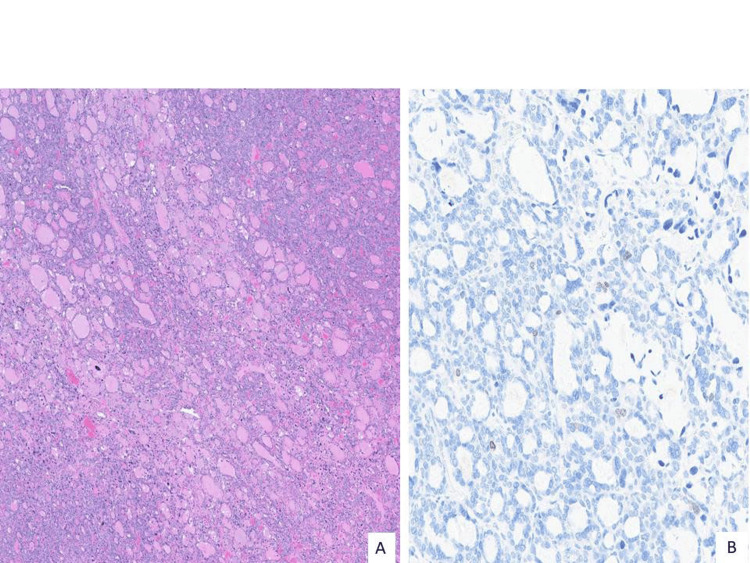
Tumor architecture and Ki-67 A. Tumor demonstrates a predominantly microfollicular architecture (H&E 20X), B. Ki-67 proliferation marker shows a low index of approximately 1-2% (Ki-67, 200X).

**Figure 4:**
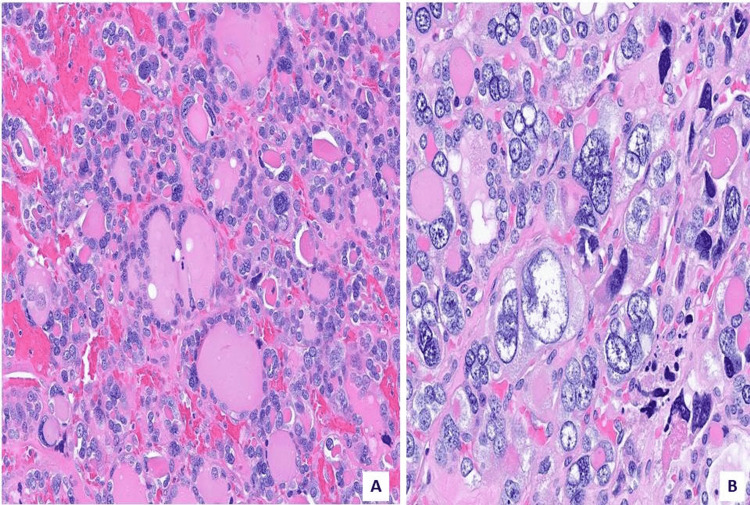
Nuclear morphology A. Pleomorphic nuclei with multinucleation (H&E 200x) and large (B.) bizarre nuclei (H&E 400X).

Gross examination of the thyroid revealed a tan-red, nodular mass that appeared confined to the thyroid except for a focally disrupted capsule and involved 95% of the left lobe. The right lobe was normal in size without a definitive tumor present grossly. Histologically, the tumor was mostly encapsulated (Figure 2), except where focally disrupted intraoperatively. There was minimal capsular invasion. Vascular invasion was not identified. Noted were areas of suspected smudged nuclear material consistent with the Azzopardi effect. The tumor demonstrated a predominantly microfollicular architecture (Figure 3A) but lacked any papillary features. The solid component was minimal to none. The nuclei were pleomorphic in size, with scattered, large, bizarre-appearing nuclei with extensive pleomorphism, multinucleation, and bizarre forms (Figure 4A, 4B). The bizarre-appearing nuclei were diffusely distributed. Despite these high-grade cytologic features, the tumor did not exhibit hallmark characteristics of poorly differentiated or anaplastic thyroid carcinoma, such as solid/trabecular/insular growth and the presence of at least one of following: at least 3 mitoses per 2 mm2, tumor necrosis, and convoluted nuclei. The Ki-67 proliferation index was low at approximately 1-2% (Figure 3B), and the mitotic rate was 1 per 2 mm2. 

Immunohistochemical staining demonstrated follicular epithelial origin (expression of TTF-1, cytokeratin 7, PAX-8, and CD56). Notably, the tumor had variable weak expression of HBME-1 and galectin-3 and was negative for synaptophysin, carcinoembryonic antigen, calcitonin, SALL4, and smooth muscle actin. FoundationOne®CDx on the resection specimen corroborated the same *DICER1 *mutation noted by the Afirma® testing. Additional negative findings included no microsatellite instability, low tumor-mutation burden, and gene mutation variants of unknown significance. The final diagnosis was rendered as “Thyroid carcinoma with follicular and anaplastic features with *DICER1 *mutation”.

Following thyroidectomy, the patient received radioactive iodine (RAI) ablation therapy with 103 mCi of I-131. A subsequent PET-CT imaging identified a pretracheal nodule and a left level-II lymph node that were biopsied and showed no evidence of malignancy. No recurrence, metastasis, or other complications have been reported to date. The patient has been under follow-up for 2.5 years.

## Discussion

We present a case of a thyroid neoplasm harboring a somatic *DICER1 *“hotspot” mutation with histological and clinical features that made classification quite challenging, given the morphologies of reported cases of thyroid tumors with *DICER1 *mutations. The large size, adherence to adjacent anatomic structures, and nuclear morphology were all features that may have signified a more aggressive tumor. However, the lack of necrosis and a low proliferation index seemed to suggest a more benign tumor. The morphology was unique for *DICER1*-associated thyroid tumors with a predominantly microfollicular architecture with bizarre/anaplastic nuclear morphology. In reviewing with other experts, it was suggested that the nuclear features, rather than indicating an anaplasia, were more indicative of “ancient change” often seen in endocrine neoplasms and peripheral nerve sheath tumors, for example. The follicular microarchitecture of this tumor was also consistent with benign neoplasms or low-grade differentiated thyroid malignancies.

The *DICER1 *gene encodes an ribonuclease (RNase) enzyme essential for the production of microRNAs (miRNAs), which serve to post-transcriptionally regulate gene expression via the silencing of target messenger RNA sequences, influencing cellular differentiation, proliferation, and apoptosis in a vast array of organs, perhaps none more so than the thyroid where it oversees follicular histogenesis and maintenance of normal function within the organ [5, 6]. Mutations of the *DICER1 *gene result in reductions of these miRNAs, eliminating the fine-tuning effect they impose on gene expression and allowing for the overexpression of oncogenes responsible for tumorigenesis and the maintenance of a microenvironment favorable to tumors, especially in the thyroid, where *DICER1 *sequelae exhibit their highest penetrance [6]. *DICER1 *mutations may be classified into two overarching forms - these being the non-heritable somatic mutations that arise de novo and usually present with fewer sequelae, and autosomal dominant germline mutations that define the DICER1 Syndrome [6, 7]. Mutations that arise in DNA sequences with the highest mutational frequency are called “hotspot mutations” and unanimously encode for defective RNase IIIa or IIIb domains [7]. 

With specific regard to thyroid tumors, a reasonable body of literature is accumulating, describing the pathological features of *DICER1*-associated thyroid tumors. Currently, the known spectrum of thyroid neoplasms associated with *DICER1 *mutations includes follicular adenomas, follicular carcinomas, papillary carcinomas (classic, follicular, and tall-cell variants), oncocytic carcinomas, poorly-differentiated thyroid carcinoma (PDTC), thyroblastomas, and ATC [4]. The case presented here, as we note, does not seem to neatly fit into the types of thyroid tumors harboring *DICER1* mutation. The rare, solid architecture and extensive nuclear pleomorphism in this case are initially concerning for PDTC. However, PDTC is defined by the Turin criteria: invasive growth pattern, solid, trabecular, or insular architecture, and harboring at least one of the following: 1) at least 3 mitoses per 2 mm2, 2) necrosis, and 3) convoluted nuclei [7,8]. Though also frequently described in the context of somatic *DICER1 *mutations, the low mitotic activity (1 per 2 mm2) and absence of necrosis in the present case do not warrant the diagnosis of PDTC [4,8]. Thyroblastoma is a highly malignant tumor with abundant atypical mitoses, a Ki-67 index often exceeding 90%, and frequent lymphovascular invasion and extrathyroidal metastases [4,9]. Though the atypical-appearing follicular cells in the present case may bear some resemblance to the primitive epithelial cells of thyroblastoma, it is important to note that immunohistochemical markers of primitive cell and mesenchymal differentiation, such as SALL4** **and smooth muscle actin, respectively, were negative here. *DICER1*-associated ATC has been reported in a single institutional review of NGS performed on thyroid neoplastic nodules [4]. The two examples in this study had concomitant differentiated carcinomas (PTC and oncocytic follicular carcinoma) adjacent to the ATC [4]. Both ATC tumors harbored *DICER1 *somatic mutations [4]. In contrast, the tumor in our case did not have delineated separate regions showing tumors with differentiated and anaplastic morphologies. The presence of a *DICER1 *mutation, in combination with “anaplastic” features but without the typical aggressive histopathological markers, may suggest that the mutation influences tumor behavior in a way that promotes cellular atypia and architectural changes without fully dedifferentiating to an aggressive phenotype. This may be reasonable given the spectrum of morphologies in *DICER1 *mutation-associated thyroid neoplasms. One hypothesis is that the *DICER1 *mutation may facilitate partial dedifferentiation from a differentiated carcinoma, such as FTC, to a state with more anaplastic characteristics [6]. However, unlike typical ATC, which displays a high proliferative index and significant necrosis, this tumor exhibited no increased mitotic activity or necrosis, indicating that the *DICER1 *mutation might be responsible for cellular atypia but not induce features considered diagnostic of PDTC or ATC.

Another possibility is that the tumor represents a “follicular carcinoma with bizarre nuclei”, a more aggressive appearing, yet still benign, counterpart of the follicular adenoma with bizarre nuclei occasionally detailed in a few case reports [10]. Much like the present case, follicular adenomas with bizarre nuclei demonstrate striking nuclei with significant pleomorphism in the absence of other high-grade features such as necrosis or abundant mitotic activity [10]. It could be conjectured that the nuclear atypia in the present case is a poorly understood degenerative artifact rather than frank dysplasia. Furthermore, the solid architecture noted in the present case was very scant and too diminutive to properly categorize this tumor as an anaplastic, poorly-differentiated, or other high-grade carcinoma. This case could indicate a carcinomatous evolution of a follicular adenoma with bizarre nuclei under the influence of *DICER1 *dysregulation.

The patient’s clinical management, including a total thyroidectomy followed by radioactive iodine (RAI) ablation, aligns with the typical approach for differentiated thyroid carcinoma with high-risk features. The lack of high-grade features such as increased mitotic activity and necrosis, combined with the benign findings from subsequent fine-needle aspiration (FNA) biopsies and imaging, suggests that the tumor may exhibit more indolent behavior than initially expected.

The *DICER1 *gene’s role in miRNA processing is critical; when mutated, it disrupts normal gene expression and can potentially drive oncogenesis. In this case, the low mitotic index and the absence of extensive necrosis or solid growth patterns deviate from the typical presentation of anaplastic thyroid carcinoma, prompting questions about the tumor’s classification and biological behavior. This case suggests that thyroid carcinomas with *DICER1 *mutations may represent a distinct subset of thyroid malignancies with unique molecular and histopathological characteristics, potentially warranting different therapeutic approaches and consideration for genetic counseling.

## Conclusions

This case emphasizes the complexity involved in diagnosing and managing thyroid tumors with mixed clinical and histological findings of benign and malignant/anaplastic. The presence of a somatic *DICER1 *mutation (p.E1705K) in a tumor with atypical histologic characteristics challenges traditional classifications of anaplastic thyroid carcinoma and suggests a unique role for *DICER1 *in modulating tumor behavior. These findings emphasize the importance of a multidisciplinary approach that includes thorough histopathological evaluation, molecular testing, and genetic counseling to provide personalized care and optimize patient outcomes. Further research is essential to elucidate the molecular mechanisms by which *DICER1 *mutations influence thyroid carcinogenesis and to assess their implications for prognosis and treatment. This case reveals the poorly understood nature of the influence of *DICER1 *mutation on thyroid differentiation, morphology, and tumorigenesis and adds to the morphological spectrum of *DICER1-*associated thyroid neoplasms.
